# Humoral and Haemocytic Responses of *Litopenaeus vannamei* to Cd Exposure

**DOI:** 10.1155/2014/903452

**Published:** 2014-05-19

**Authors:** Juan C. Bautista-Covarrubias, Germán J. Velarde-Montes, Domenico Voltolina, Luz M. García-de la Parra, Martín F. Soto-Jiménez, Martín G. Frías-Espericueta

**Affiliations:** ^1^Programa Regional del Posgrado en Biotecnología, Facultad de Ciencias Químico Biológicas, Universidad Autónoma de Sinaloa, 80000 Culiacán, Mexico; ^2^Secretaría de Investigación y Posgrado, Universidad Autónoma de Nayarit, Boulevard Tepic-Xalisco S/N, 63000 Tepic, Nayarit, Mexico; ^3^Laboratorio de Estudios Ambientales, Facultad de Ciencias del Mar, Universidad Autónoma de Sinaloa, AP 1132, 82000 Mazatlán, Sinaloa, Mexico; ^4^Laboratorio UAS-CIBNOR, Centro de Investigaciones Biológicas del Noroeste, P.O. Box 1132, 82000 Mazatlán, Sinaloa, Mexico; ^5^Centro de Investigación en Alimentación y Desarrollo, Unidad Mazatlán, Avenida Sábalo-Cerritos S/N, 82010 Mazatlán, Sinaloa, Mexico; ^6^Instituto de Ciencias del Mar y Limnología, Unidad Académica Mazatlán, Universidad Nacional Autónoma de México, P.O. Box 811, 82000 Mazatlán, Sinaloa, Mexico

## Abstract

White shrimp, *Litopenaeus vannamei*, subadults were exposed to four dilutions of the 96 h cadmium LC_50_ reported for postlarvae (PL12) of this species, and the effects were evaluated after 5, 48, and 96 h of exposure. While treatments did not affect survival and hemolymph clotting time increased with time, but not as a response to Cd exposure, the intensity of other responses was related to concentration, to time of exposure, and to their interaction. Hemocyanin decreased with time in all metal concentrations but increased in the control treatment, and an almost similar trend was observed with hemocyte numbers. As an initial response, phenoloxidase activity decreased with all metal concentrations, but it increased later to values similar or higher than the control treatment.

## 1. Introduction

The coastal environments of the Mexican Pacific receive metal contaminants from multiple sources, such as the several rivers flowing from important mining districts [1] and the waterways and canals draining the fertile coastal plains of the states of Sonora and Sinaloa, which support highly intensive agricultural activities [2, 3]. Because of this, the metal contents of the marine biota are under continuous scrutiny [4, 5], and among these the levels of cadmium detected in different aquatic organisms of the coastal environments of NW Mexico indicate that this metal might become of concern for aquatic life and possibly for human health [6].

Cadmium has several detrimental effects in aquatic organisms, since it affects respiration and osmoregulation because it causes structural and physiological damages to gills [7, 8]. In crustaceans, it also causes increased secretion of the gonad-inhibiting hormone (GIH) and alteration of calcium homeostasis and DNA damage [9–12]. Additionally, exposure to Cd increases the generation of reactive oxygen species (ROS) and induces apoptosis or necrosis of immune cells, and this imbalance between ROS generation and antioxidant defense mechanisms causes increased oxidative stress [13, 14].

In view of these alterations of the defense mechanisms of aquatic invertebrates, which consist of cellular and humoral responses, the aim of this study was to determine the effect on survival, clotting time, hemocyanin, number of hemocytes, and phenoloxidase activity of* Litopenaeus vannamei* juveniles exposed to four sublethal concentrations of cadmium.

## 2. Material and Methods

The experimental Cd concentrations were 8.8, 24.9, 249, and 1245 *μ*g L^−1^. The first is the safe concentration (continuous concentration criterion: CCC) for continuous exposure of marine life to Cd [15] and is equivalent to 0.35% of the LC_50_-96 h (2.49 mg L^−1^) for shrimp postlarvae (wet weight, 005–0.08 mg) exposed to water-borne Cd [16]. The three remaining are 1, 10, and 50% dilutions of the LC_50_-96 h.

The size of* Litopenaeus vannamei* juveniles (mean total length 11.9 ± 1.2 cm and mean wet weight 10.1 ± 3.3 g) was chosen to ensure that, in view of the large difference in weight, these Cd levels were unlikely to cause any mortality but could affect the activation of humoral and cellular defense responses, since cadmium toxicity is mainly due to oxidative stress.

Shrimp juveniles (200) obtained from a local shrimp farm were held in two holding tanks for three days and acclimated for three additional days to the conditions of the experiment in ten 40 L (60 × 40 × 33 cm) aquaria with 15 shrimp per tank [17]. This concentration, equivalent to 62.5 shrimp/m^2^, was chosen as representative of the stressing conditions of intensive commercial shrimp cultures, although it has been shown that, even without water exchanges, growth and survival in these cultures may be normal for between two and three months [18]. During acclimation, shrimps were fed* ad libitum* 35% protein commercial feed (Vimifos) three times a day. Unconsumed food was removed with a siphon after 1 hour. Holding conditions were temperature 20 ± 1°C, salinity 34 ± 1, oxygen 5 ± 1 mg L^−1^, 12 h : 12 h light-dark photoperiod, and daily water renovations (80%) using 10 *μ*m-filtered, UV-treated seawater.

On the fourth day, the test solutions were added to the experimental aquaria using the appropriate volumes and dilutions of a stock solution prepared with CdCl_2_ (Baker, GR grade) dissolved in double distilled water to obtain a concentration of 1,000 mg Cd L^−1^. All treatments were in duplicate (two 15-shrimp groups for each concentration), including two control aquaria with no Cd. Feeding ceased 24 h before the experiment and shrimps remained unfed throughout the experiment, which lasted 96 h.

Hemolymph samples were obtained after 5, 48, and 96 hours from Cd addition, using three shrimps in premoult stage C [19] from each of the 10 aquaria (six shrimps for each concentration). Samples were extracted from the ventral part of the first abdominal segment with an insulin syringe. Immediately after extraction, clotting time was determined at 22 ± 1°C with the capillary method, using 20 *μ*L of hemolymph drawn into a 1.15 mm internal diameter capillary tube, which was inverted continuously until the hemolymph ceased flowing. The initial time was that of needle insertion [20].

Hemocyanin was quantified at 335 nm in duplicate for each shrimp in 1 : 100 diluted hemolymph : distilled water sample [21]. Total hemocyte count (THC) was performed on 50 *μ*L aliquots of 1 : 1 (V/V) dilutions of hemolymph with the anticoagulant described in [22] (27 mM trisodium citrate, 385 mM NaCl, 115 mM glucose, and pH 6.6) and diluted further with formalin (4% and 0.45 M NaCl) to a final 1 : 4 dilution. Counts were performed with a Neubauer chamber at 400X, using the four corner quadrants and an additional one, selected at random [23].

Phenoloxidase (PO) activity was determined in triplicate in a microplate adding 30 *μ*L cacodylate buffer to 30 *μ*L of hemocyte lysate obtained with an ultrasound generator [24]. After 10 minutes at 25°C, 170 *μ*L of L-DOPA (L, 3,4-dihydroxyphenylalanine) was added and the absorbance was read after 10 minutes at 492 nm in a Multiskan Ascent 354 Labsystems plate reader [25]. Values were adjusted to relative units (PO units mg^−1^ of protein), using the protein concentration of the sample determined with the Bradford method [26], using BioRad reagents and bovine serum albumin as standard.

Data were not normal and/or homoscedastic (Kolmogorov-Smirnov and Bartlett's tests, *P* < 0.05). Therefore the mean values obtained were compared using two-way ANOVA and Holm-Sidak's tests after rank transformation of the original data [27, 28], with *α* = 0.05 for all tests.

## 3. Results and Discussion

Survival was 100% in all experimental aquaria. Clotting time tended to decrease with time of exposure in all treatments, which was the only significant (*P* < 0.01) source of variation. In most cases values were significantly (*P* < 0.05) lower after 96 than after 5 h of exposure, and there were no significant differences between control and Cd treatments (Table 1).

Since the clotting process is an essential defense response for crustaceans, an increase of the normal clotting time should be taken as an indication of an impaired (slower) response to the presence of a stressing agent [14, 29, 30]. Therefore, the lack of difference between control and Cd-exposed organisms indicates that the Cd concentrations were below the level needed for its activation, while the clear tendency to shorter clotting times with increasing time of exposure seems to indicate a progressive acclimation to the stressor represented by the high population densities in the experimental aquaria.

Cd concentration, time of exposure, and their interaction were significant (*P* < 0.01) sources of variation for all remaining variables used to evaluate the effect of shrimp exposure to Cd. Hemocyanin is the main respiratory pigment of crustaceans, it represents 60 to 93% of the total protein content of their hemolymph [31] and its concentration has been related to exposure to different stressors [32]. In this work the mean values determined after 5 h in Cd-exposed shrimps were significantly (*P* < 0.05) higher than those of control shrimps and were independent from Cd concentrations. However, values increased significantly in the control shrimps after 48 h and 96 hours, while they tended to decrease with time of exposure in all Cd concentrations. By the end of the experiment all values of Cd-exposed shrimps were significantly lower than after 5 h, and the lowest values were those determined with 24.9 and 1245 *μ*g L^−1^ Cd (Table 2A).

This trend parallels that of hemolymph protein contents (Table 2B), and both seem to indicate a short-time response to Cd exposure. After five hours, hemocyanin and proteins were close to twice the concentration of the control shrimp, which coincides with the trend observed in shrimp exposed to handling stress [33] and indicates an initial mobilization of proteins to satisfy the high initial energy demand following stress exposure [34], while the low value found after 96 h with the highest Cd concentration seems to indicate that the energy demand was higher in this case and that the proteins used to satisfy the energy demand caused by the long-term exposure to a continuing situation of stress could not be replenished, in view of the fact that shrimps remained unfed through the experiment.

There were no time-related differences between the mean hemocyte concentrations of the control shrimps, whereas in all Cd treatments there was a clear tendency to decrease with time of exposure, although differences between 5 and 96 h were not significant for the treatment with 24.9 *μ*g L^−1^ Cd. With one exception (5 hours, 249 *μ*g L^−1^ Cd), the values determined for all times of exposure in the four Cd treatments were significantly (*P* < 0.05) lower than those of the control shrimps, and the lowest mean value after 96 h was that determined for the highest concentration of Cd (Table 3).

Cadmium is known to induce apoptosis and/or cell necrosis in a variety of cells and organisms [35–39]. This may explain the tendency to the progressively decreasing hemocyte concentrations registered in the Cd treatments and is in agreement with the tendency determined in the crab* Sinopotamon henanense* exposed to Cd concentrations equivalent to between 3.1 and 50% of the LC50 of this species, which caused hemocyte apoptosis and levels of necrosis to progressively increase, in direct relation to the level of Cd concentration [14, 40].

Phenoloxidase (PO) activity was significantly higher in the control shrimps than in all Cd treatments after 5 h of exposure, but later activities varied with different treatments: it was unchanged after 48 h in the control shrimps, whereas all Cd treatments showed a significant increase. As a result, there were no differences between control and Cd-exposed shrimps, with the exception of the treatment with 8.8 *μ*g L^−1^, which at this time had the significantly highest value. After 96 h PO activity increased significantly in the control shrimps and in those exposed to 24.9 *μ*g L^−1^ Cd, whereas it decreased in the remaining treatments (Table 4A). However, PO activity is given as the ratio of PO units to plasma protein, which showed significant changes as a function of Cd levels, of the time of sampling (including control shrimps) and of their interaction. In view of the positive relation between plasma protein and PO concentrations [41], this probably caused at least a part of the changes of PO activity found in this work.

The proPo system is localized in the granules of semigranular and granular hemocytic cells which, once stimulated, release phenoloxidase to plasma. For this reason, we calculated the ratio of PO activity to plasma cell numbers, which gave a fairly different picture to that described using the PO to protein ratio. In this case, there was a common tendency to increase as a response to increasing time of exposure. In all cases, the 96 h values were significantly higher than after 5 hours, indicating that the continuous stimulation of the immune system caused by the presence of cadmium resulted in an increased PO production by a continuously decreasing number of plasma cells (Table 4B).

## 4. Conclusions

Toxic metals in the environment may affect the organism's capacity of adequate defense responses even when their concentrations are below the level of risk. This was determined in experiments with* L. vannamei *exposed to levels of Cd which are supposed to be safe for long-term exposure to this metal. These caused low hemocyanin and plasma protein concentrations and decreased hemocytes numbers, eventually leading to increased susceptibility to diseases of the organisms exposed continuously to metal stress.

## Figures and Tables

**Table 1:** Mean values (±SD) of hemolymph clotting time (minutes) of *Litopenaeus vannamei* exposed to four concentrations (*μ*g L^−1^) of cadmium.

Time (hours)	Control	8.8	24.9	249	1245
5	2.49 ± 1.24^b^	2.17 ± 0.26^b^	2.74 ± 0.47^b^	2.97 ± 0.63^b^	2.38 ± 0.65^b^
48	2.23 ± 0.53^b^	2.13 ± 0.17^b^	2.09 ± 0.12^b^	2.24 ± 0.69^ab^	2.18 ± 0.51^b^
96	1.62 ± 0.28^a^	1.77 ± 0.15^ab^	1.55 ± 0.28^a^	1.83 ± 0.78^a^	1.99 ± 0.72^ab^

Equal or common letters indicate lack of significant differences (two-way ANOVA and Holm-Sidak test, a ≤ ab ≤ b and a < b).

**Table 2:** Mean (±SD) hemocyanin (A: mmol L^−1^) and protein (B) concentrations (mg mL^−1^) of *L. vannamei* exposed to four concentrations (*μ*g L^−1^) of cadmium.

Time (hours)	Control	8.8	24.9	249	1245
A					
5	0.42 ± 0.12^a^	0.96 ± 0.24^cd^	0.95 ± 0.13^cd^	1.14 ± 0.57^d^	0.93 ± 0.13^cd^
48	0.88 ± 0.18^bc^	0.73 ± 0.08^b^	0.83 ± 0.10^bc^	0.83 ± 0.09^c^	0.83 ± 0.06^bc^
96	0.78 ± 0.23^b^	0.78 ± 0.13^b^	0.61 ± 0.06^a^	0.72 ± 0.17^ab^	0.60 ± 0.18^a^

B					
5	15.53 ± 4.94^c^	21.91 ± 3.02^d^	22.81 ± 3.35^d^	20.54 ± 7.71^cd^	16.99 ± 5.23^cd^
48	15.10 ± 5.29^c^	11.11 ± 7.68^b^	13.64 ± 5.86^bc^	13.93 ± 5.37^b^	12.30 ± 4.10^b^
96	14.67 ± 7.58^c^	14.50 ± 6.57^c^	13.43 ± 5.65^bc^	14.86 ± 2.72^bc^	7.69 ± 7.03^a^

Equal or common letters indicate lack of significant differences (two-way ANOVA and Holm-Sidak tests, a ≤ ab ≤ b ≤ bc ≤ cd and a < b < c < d).

**Table 3:** Mean values (±SD) of total hemocyte concentrations (10^6^ cells mL^−1^) of *L. vannamei* exposed to four concentrations (*μ*g L^−1^) of cadmium.

Time (hours)	Control	8.8	24.9	249	1245
5	6.06 ± 2.29^d^	3.93 ± 2.04^c^	4.58 ± 3.53^c^	5.88 ± 3.26^cd^	5.38 ± 3.72^c^
48	7.80 ± 3.63^d^	2.84 ± 1.45^c^	4.21 ± 2.82^c^	3.38 ± 2.35^c^	2.88 ± 1.71^c^
96	5.84 ± 4.66^d^	1.60 ± 1.04^b^	3.44 ± 3.16^c^	1.59 ± 0.76^b^	1.25 ± 1.14^a^

Equal or common letters indicate lack of significant differences (two-way ANOVA and Holm-Sidak test, a ≤ ab ≤ b ≤ bc ≤ cd and a < b < c < d).

**Table 4:** Mean values (±SD) of phenoloxidase activity (A), as PO units mg protein^−1^, and of phenoloxidase activity to hemocyte concentration ratios (B) in *L. vannamei*, exposed to four concentrations (*μ*g L^−1^) of cadmium.

Time (hours)	Control	8.8	24.9	249	1245
A					
5	0.107 ± 0.010^b^	0.076 ± 0.010^a^	0.068 ± 0.007^a^	0.072 ± 0.011^a^	0.085 ± 0.020^a^
48	0.101 ± 0.007^b^	0.152 ± 0.020^d^	0.101 ± 0.013^b^	0.113 ± 0.047^b^	0.104 ± 0.012^b^
96	0.121 ± 0.022^c^	0.112 ± 0.007^bc^	0.138 ± 0.012^d^	0.088 ± 0.041^b^	0.109 ± 0.012^b^

B					
5	0.080 ± 0.023^a^	0.123 ± 0.141^ab^	0.158 ± 0.213^ab^	0.064 ± 0.036^a^	0.164 ± 0.148^ab^
48	0.059 ± 0.021^a^	0.280 ± 0.174^bc^	0.155 ± 0.117^abc^	0.209 ± 0.154^abc^	0.179 ± 0.089^ab^
96	0.123 ± 0.075^ab^	0.327 ± 0.153^cd^	0.269 ± 0.154^cd^	0.414 ± 0.152^cd^	0.683 ± 0.678^d^

Equal or common letters indicate lack of significant differences (two-way ANOVA and Holm-Sidak test, a ≤ ab ≤ b ≤ bc ≤ cd and a < b < c < d).

## References

[1] Frías-Espericueta MG, Mejía-Cruz R, Osuna López I (2014). Metal discharges by Sinaloa rivers to the coastal zone of NW Mexico. *Bulletin of Environmental Contamination and Toxicology*.

[2] Green-Ruiz C, Páez-Osuna F (2003). Heavy metal distribution in surface sediments from a subtropical coastal lagoon system associated with an agricultural basin. *Bulletin of Environmental Contamination and Toxicology*.

[3] Ochoa-Valenzuela LE, Gómez-Álvarez A, García-Rico L, Villalba-Atondo AI (2009). Distribution of heavy metals in surface sediments of the Bacochibampo Bay, Sonora, Mexico. *Chemical Speciation and Bioavailability*.

[4] Frías-Espericueta MG, Osuna-López JI, Izaguirre-Fierro G, Aguilar-Juárez M, Voltolina D (2010). Cadmio y plomo en organismos de importancia comercial de la zona costera de Sinaloa, México: 20 años de estudios. *Oceánides*.

[5] Ruelas-Inzunza J, Green-Ruiz C, Zavala-Nevárez M, Soto-Jiménez M (2011). Biomonitoring of Cd, Cr, Hg and Pb in the Baluarte River basin associated to a mining area (NW Mexico). *Science of the Total Environment*.

[6] Frías-Espericueta MG, Osuna-López I, Voltolina D (2009). The contents of Cd, Cu, Pb and Zn of the white shrimp *Litopenaeus vannamei* (Boone, 1931) of six coastal lagoons of Sinaloa, NW Mexico. *Revista de Biologia Marina y Oceanografia*.

[7] Soegianto A, Charmantier-Daures M, Trilles JP, Charmantier G (1999). Impact of cadmium on the structure of gills and epipodites of the shrimp *Penaeus japonicus* (Crustacea: Decapoda). *Aquatic Living Resources*.

[8] Soegianto A, Winami D, Handayani US, Hartati (2013). Bioaccumulation, elimination, and toxic effect of cadmium on structure of gills and hepatopancreas of freshwater prawn *Macrobrachium sintangese* (De Man, 1898). *Water, Air, & Soil Pollution*.

[9] Revathi P, Vasanthi LA, Munuswamy N (2011). Effect of cadmium on the ovarian development in the freshwater prawn *Macrobrachium rosenbergii* (De Man). *Ecotoxicology and Environmental Safety*.

[10] Rodríguez EM, López Greco LS, Fingerman M (2000). Inhibition of ovarian growth by cadmium in the fiddler crab, *Uca pugilator* (Decapoda, Ocypodidae). *Ecotoxicology and Environmental Safety*.

[11] Lee R, Kim GB (2002). Comet assays to assess DNA damage and repair in grass shrimp embryos exposed to phototoxicants. *Marine Environmental Research*.

[12] Chang M, Wang WN, Wang AL (2009). Effects of cadmium on respiratory burst, intracellular Ca^2+^ and DNA damage in the white shrimp *Litopenaeus vannamei*. *Comparative Biochemistry and Physiology C: Toxicology and Pharmacology*.

[13] Lei W, Wang L, Liu D, Xu T, Luo J (2011). Histopathological and biochemical alternations of the heart induced by acute cadmium exposure in the freshwater crab *Sinopotamon yangtsekiense*. *Chemosphere*.

[14] Wang J, Zhang P, Shen Q (2013). The effects of cadmium exposure on the oxidative state and cell death in the gill of freshwater crab *Sinopotamon henanense*. *PLoS ONE*.

[15] EPA (2009). *National Recommended Water Quality Criteria*.

[16] Frías-Espericueta MG, Voltolina D, Osuna-López JI (2001). Acute toxicity of cadmium, mercury, and lead to whiteleg shrimp (*Litopenaeus vannamei*) postlarvae. *Bulletin of Environmental Contamination and Toxicology*.

[17] Frías-Espericueta MG, Abad-Rosales S, Nevárez-Velázquez AC (2008). Histological effects of a combination of heavy metals on Pacific white shrimp *Litopenaeus vannamei* juveniles. *Aquatic Toxicology*.

[18] Hopkins JS, Hamilton RD, Sandier PA, Browdy CL, Stokes AD (1993). Effect of water exchange rate on production, water quality, effluent characteristics and nitrogen budgets of intensive shrimp ponds. *Journal of the World Aquaculture Society*.

[19] Robertson L, Bray W, Leung-Trujillo J, Lawrence A (1987). Practical molt staging of *Penaeus setiferus* and *Penaeus stylirostris*. *Journal of the World Aquaculture Society*.

[20] Jussila J, McBride S, Jago J, Evans LH (2001). Hemolymph clotting time as an indicator of stress in western rock lobster (*Panulirus cygnus* George). *Aquaculture*.

[21] Pascual C, Sánchez A, Zenteno E (2006). Biochemical, physiological, and immunological changes during starvation in juveniles of *Litopenaeus vannamei*. *Aquaculture*.

[22] Huang J, Yang Y, Wang A (2010). Reconsideration of phenoloxidase activity determination in white shrimp *Litopenaeus vannamei*. *Fish and Shellfish Immunology*.

[23] Arredondo-Vega BO, Voltolina D, Arredondo-Vega BO, Voltolina D (2007). Concentración, recuento celular y tasa de crecimiento. *Métodos y Herramientas Analíticas en la Evaluación de la Biomasa Microalgal*.

[24] Ji PF, Yao CL, Wang ZY (2009). Immune response and gene expression in shrimp (*Litopenaeus vannamei*) hemocytes and hepatopancreas against some pathogen-associated molecular patterns. *Fish and Shellfish Immunology*.

[25] Hernández-López J, Gollas-Galvan T, Vargas-Albores F (1996). Activation of the prophenoloxidase system of the brown shrimp (*Penaeus californiensis* Holmes). *Comparative Biochemical Physiology*.

[26] Bradford MM (1976). A rapid and sensitive method for the quantitation of microgram quantities of protein utilizing the principle of protein dye binding. *Analytical Biochemistry*.

[27] Iman RL (1974). A power study of a rank transform for the two-way classification model when interaction may be present. *Canadian Journal of Statistics*.

[28] Conover WJ, Iman RL (1981). Rank transformations as a bridge between parametric and nonparametric statistics. *The American Statistician*.

[29] Song YL, Yu CI, Lien TW, Huang CC, Lin MN (2003). Haemolymph parameters of Pacific white shrimp (*Litopenaeus vannamei*) infected with Taura syndrome virus. *Fish and Shellfish Immunology*.

[30] Yoganandhan K, Thirupathi S, Sahul Hameed AS (2003). Biochemical, physiological and hematological changes in white spot syndrome virus-infected shrimp, *Penaeus indicus*. *Aquaculture*.

[31] Cheng W, Liu C-H, Yan D-F, Chen J-C (2002). Hemolymph oxyhemocyanin, protein, osmolality and electrolyte levels of whiteleg shrimp *Litopenaeus vannamei* in relation to size and molt stage. *Aquaculture*.

[32] Bachère E, Gueguen Y, Gonzalez M, de Lorgeril J, Garnier J, Romestand B (2004). Insights into the anti-microbial defense of marine invertebrates: the penaeid shrimps and the oyster *Crassostrea gigas*. *Immunological Reviews*.

[33] Mercier L, Racotta IS, Yepiz-Plascencia G (2009). Effect of diets containing different levels of highly unsaturated fatty acids on physiological and immune responses in Pacific whiteleg shrimp *Litopenaeus vannamei* (Boone) exposed to handling stress. *Aquaculture Research*.

[34] Racotta IS, Palacios E (1998). Hemolymph metabolic variables in response to experimental manipulation stress and serotonin injection in *Penaeus vannamei*. *Journal of the World Aquaculture Society*.

[35] Hamada T, Tanimoto A, Sasaguri Y (1997). Apoptosis induced by cadmium. *Apoptosis*.

[36] Lohmann RD, Beyersmann D (1993). Cadmium and zinc mediated changes of the Ca^2+^-dependent endonuclease in apoptosis. *Biochemical and Biophysical Research Communications*.

[37] Habeebu SSM, Liu J, Klaassen CD (1998). Cadmium-induced apoptosis in mouse liver. *Toxicology and Applied Pharmacology*.

[38] Sokolova IM, Evans S, Hughes FM (2004). Cadmium-induced apoptosis in oyster hemocytes involves disturbance of cellular energy balance but no mitochondrial permeability transition. *The Journal of Experimental Biology*.

[39] Agnello M, Filosto S, Scudiero R, Rinaldi AM, Roccheri MC (2007). Cadmium induces an apoptotic response in sea urchin embryos. *Cell Stress and Chaperones*.

[40] Qin Q, Qin S, Wang L, Lei W (2012). Immune responses and ultrastructural changes of hemocytes in freshwater crab *Sinopotamon henanense* exposed to elevated cadmium. *Aquatic Toxicology*.

[41] Verdú JR, Casas JL, Cortez V, Gallego B, Lobo JM (2013). Acorn consumption improves the immune response of the dung beetle *Thorectes lusitanicus*. *PLoS ONE*.

